# Clinical risk factors and outcomes of young patients with acute ST segment elevation myocardial infarction: a retrospective study

**DOI:** 10.1186/s12872-023-03392-8

**Published:** 2023-07-17

**Authors:** Ming-Ting Liang, Ying Pang, Li-Li Gao, Li-Jin Han, Heng-Chen Yao

**Affiliations:** 1 Cheeloo College of Medicine, Shandong University, Jinan, Shandong China; 2 Department of Cardiology, Liaocheng People’s Hospital, Shandong University, Liaocheng, 252000 Shandong China; 3 Department of Nursing, Liaocheng People’s Hospital, Liaocheng, Shandong China

**Keywords:** Acute ST segment myocardial infarction, Young people, Risk factors

## Abstract

**Background:**

This study aimed to analysis the clinical characteristics and prognosis of acute STEMI in patients aged ≤ 45 years.

**Methods:**

Seven hundred and one patients with STEMI from Liaocheng People’s Hospital from January 2018 to March 2021 were included in this study. Clinical characteristics, management, and outcomes (average follow-up: 11.5 months) were compared between patients aged ≤ 45 years and those aged > 45 years.

**Results:**

Of the patients with STEMI who underwent primary percutaneous coronary intervention, 108 (15.4%) were aged ≤ 45 years. Compared to the older group, the younger patient group included more males, current smokers, and those with alcohol use disorder (AUD) or a family history of ischaemic heart disease (IHD). The culprit vessel in young patients was the left anterior descending (LAD) artery (60% vs. 45.9%, P = 0.031), which may have been due to smoking (odds ratio, 3.5; 95% confidence interval: 1.12–10.98, P = 0.042). Additionally, young patients presented with higher low-density lipoprotein and lower high-density lipoprotein levels than older patients; uric acid levels were also significantly higher in younger patients than that in the older group. Diabetes showed a trend toward major adverse cardiovascular events (MACE) in both groups; age and sex were both independent predictors of MACE in older patients.

**Conclusion:**

More patients who were smokers, had AUD, or a family history of IHD were present in the young patient group. Hyperuricaemia (but not dyslipidaemia) was a prevalent risk factor in patients aged ≤ 45 years. Diabetes should be controlled to reduce cardiovascular events in young patients.

## Introduction

Although the incidence of acute myocardial infarction (AMI) is declining in developed countries, AMI remains one of the main cardiovascular diseases with a heavy burden worldwide [1, 2]. As the largest developing country in the world, China has been recording an increasing amount of mortality due to AMI annually [3]. Young adults make up a considerable percentage of AMI hospitalizations in many countries, possibly because of the rising prevalence of conventional cardiovascular disease risk factors such as smoking, hypertension, dyslipidaemia, and diabetes among young adults [4, 5]. Young Chinese patients with AMI may have a different risk factor profile and long-term clinical outcomes compared with young patients in the West, especially acute ST-segment myocardial infarction (STEMI), which is directly related to heart failure and sudden death, [6, 7] in addition, Young people especially in the northern regions of China eat more stir fried vegetables and salty food, have a high proportion of alcohol consumption, which were related to cardiovascular disease [8, 9]. While previous studies have described the characteristics and outcomes of young patients with MI, there is a lack of contemporary data among young China patients with STEMI who are treated with PPCI. The study of these patients may have novel implications in understanding the status and quality of STEMI management.

This study aimed to explore the clinical profile, serum index, coronary angiography, treatment, and long-term prognostic factors of STEMI among Chinese patients aged ≤ 45 years and those aged > 45 years and to provide evidence for the prevention of STEMI in this young Chinese population.

## Methods

### Study population

This was a retrospective study of patients with acute STEMI who presented within 12 h of symptom onset and underwent PCI during their initial hospitalization at the Liaocheng People’s Hospital, China from January 2019 to March 2021, using the Third Universal Definition of Myocardial Infarction [10]. Only patients with type 1 myocardial infarction on index admission were included.

This study excluded patients with chronic hepatic and renal insufficiency, chronic obstructive pulmonary disease, malignant tumors, hematological diseases, acute inflammation or infectious diseases, immunodeficiency diseases.

### Risk factors and data collection

Risk factors (hypertension, diabetes mellitus, smoking, alcohol use disorder [AUD], and a family history of ischaemic heart disease [IHD̄̄̄]) and serum indices were ascertained through a detailed review of electronic medical records, such as the red cell distribution width (RDW), platelet distribution width (PDW), ratio of lymphocytes to neutrophils (NLRs), C-reactive protein (CRP) test, NT-proBNP, triglyceride (TG), total cholesterol (TC), low-density lipoprotein (LDL), high-density lipoprotein (HDL), lipoprotein a (Lpa), random blood sugar, homocysteine (Hcy), and uric acid (UA).

### Angiographic findings and treatment

Coronary artery stenosis was defined as a greater than 50% reduction in the lumen diameter of any of the three coronary arteries or their primary branches. The culprit vessel and number of multi-vascular diseases were obtained by coronary angiography.

Based on STEMI management guidelines, all patients with STEMI received antipyretics (aspirin and clopidogrel/ticagrelor), statins, β-blockers, angiotensin-converting enzyme inhibitors (ACEI), and angiotensin receptor blockers (ARBs)/ Angiotensin receptor-neprilysin inhibitors (ARNIs).

### Follow up

Patients who survived and were discharged from the hospital were followed up using telephone calls or clinical visits at 30 days, 6, 12, 18, and 24 months. The median follow up period was 11.5 months. Major adverse cardiovascular events (MACE), including death, recurrent myocardial infarction, stroke, rehospitalisation for heart failure, and rejuvenation due to angina, were recorded.

### Statistical analysis

According to data distribution, continuous variables are presented as means ± standard deviation or medians (quartiles) and were compared using the student’s t-test or non-parametric statistical test. Categorical variables are reported as frequencies (percentage) and were compared using the likelihood ratio χ^2^ or Fisher exact test, as appropriate. Long-term prognostic predictors were calculated using multivariable logistic regression models. The Mann–Whitney U test was used for non-normally distributed variables. All statistical analyses were performed using SPSS version 26 software. Statistical significance was set at P < 0.05.

## Results

### Baseline characteristics and risk factors

The baseline characteristics of the patients are presented in Table 1. The mean ages of patients in the young (≤ 45 years) and older (> 45 years) patient groups were 39 ± 4.3 and 65 ± 9.7 years, respectively (p < 0.001). Compared with the older patient group, the younger patient group included more males (98.1% vs. 71.8%, p < 0.001), current smokers (76.9% vs. 50.6%, p < 0.001), and patients with AUD (63.9% vs. 28.2%, p < 0.001) or a family history of IHD (17.6% vs. 8.9%, p = 0.025). The prevalence of hypertension and diabetes mellitus in young patients was significantly lower than that in older patients (35.2% vs. 57.2% and 11.1% vs. 31.7%, respectively, p < 0.001).

The level of PDW in younger patients was higher than that in older patients (11.69 ± 2.10 vs. 11.19 ± 1.90, p = 0.032) (Table 2), while the levels of RDW and NLRs were lower (p < 0.05). In addition, the NT-proBNP level of the older group was significantly higher than that of the younger group (1150.13 ± 1810.69 vs. 853.79 ± 856.38, p = 0.018). Compared with older patients, dyslipidaemia was more common in younger patients, including higher levels of TG, TC, and LDL; HDL was lacking in them (all p > 0.05). However, there was no difference in Lp(a) and hcy levels between the two groups. Furthermore, uric acid levels were higher in the younger patient group than in the older group (371.06 ± 100.47 vs. 295.31 ± 84.14, p < 0.001).

**Table 1:** Baseline characteristics of patients in different age groups

Variables	Age ≤ 45 years (n = 108)	age > 45 years (n = 593)	P value
Hypertension	38(35.2)	339(57.2)	< 0.001
Diabetes mellitus	12(11.1)	188(31.7)	< 0.001
Current smoker	83(76.9)	300(50.6)	< 0.001
Alcohol use disorder	69(63.9)	167(28.2)	< 0.001
Family history of IHD	19(17.6)	53(8.9)	0.025
Age (years)	39 ± 4.3	65 ± 9.7	< 0.001
Sex(female)	2(1.9)	167(28.2)	< 0.001

IHD, ischemic heart disease

**Table 2:** Laboratory test indicators of patients in the two groups

Variables	Age ≤ 45 years(n = 108)	Age > 45 years (n = 593)	P value
Red cell distribution width(fL)	41.87 ± 3.85	42.76 ± 3.27	0.027
Platelet distribution width(fL)	11.69 ± 2.10	11.19 ± 1.90	0.032
The ratio of lymphocytes to neutrophils	4.54 ± 3.71	5.99 ± 5.03	0.014
CRP(mg/L)	8.99 ± 12.27	14.13 ± 78.67	0.566
NT-proBNP(pg/ml)	853.79 ± 856.38	1150.13 ± 1810.69	0.018
Triglyceride(mmol/L)	2.44 ± 1.81	1.74 ± 1.25	0.001
Total cholesterol(mmol/L)	5.06 ± 1.35	4.65 ± 1.10	0.010
LDL(mmol/L)	3.23 ± 0.95	2.82 ± 0.81	0.000
HDL(mmol/L)	1.04 ± 0.27	1.12 ± 0.39	0.028
Lipoprotein a(mg/L)	253.12 ± 277.47	268.01 ± 279.17	0.681
Random blood sugar(mmol/L)	7.27 ± 5.06	8.82 ± 3.50	0.001
Homocysteine (umol/L)	16.10 ± 10.71	13.90 ± 8.67	0.112
Uric Acid (umol/L)	371.06 ± 100.47	295.31 ± 84.14	0.000

CRP, C-reactive protein; LDL, low-density lipoprotein; HDL, high-density lipoprotein

### Angiographic characteristics and treatment

More young patients with STEMI presented with the left anterior descending (LAD) coronary artery as the culprit vessel (60.2% vs. 45.9%, p = 0.031) (Table 3), which may have been due to smoking (odds ratio (OR): 3.5, 95% confidence interval (CI) 1.12–10.98, p = 0.042). However, the culprit vessel was always the right coronary artery in older patients (24.1% vs. 41.1%, p = 0.004), and no statistical differences were observed for the left circumflex artery in the two groups. Multi-vessel disease was less prevalent in younger patients with STEMI than in older patients (two-vessel disease: 22.2% vs. 38.1%, p = 0.001; three-vessel disease: 12.9% vs. 26.1%, p = 0.011).

**Table 3:** Angiographic characteristics and drugs used in patients in the two groups

Variables	Age ≤ 45 years(108)	Age > 45 years(593)	P value
Culprit vessel			
Left anterior descending artery	65(60.2)	272(45.9)	0.031
Left circumflex artery	12(11.1)	63(10.6)	0.845
Right coronary artery	26(24.1)	244(41.1)	0.004
Two vessel simultaneous	3(2.7)	11(1.9)	0.655
Left main coronary artery	0	3(0.5)	
Number of vessel disease			
2-vessel disease	24(22.2)	226(38.1)	0.001
3-vessel disease	14(12.9)	155(26.1)	0.011
Antiplatelet therapy			
Ticagrelor	91(84.3)	481(81.1)	0.536
Clopidogrel	16(14.8)	112(18.9)	0.526
Rivaroxaban and Clopidogrel	0	7(1.2)	
Statins	101(93.5)	581(97.9)	0.038
β blockers	100(92.6)	475(80.1)	0.008
ACEI	54(50)	241(40.6)	0.142
ARB	25(23.1)	136(22.9)	1
ARNI	4(3.7)	37(6.2)	0.788
ACEI/ARB	82(75.9)	422(71.2)	0.891
Death in hospital	2(1.9)	17(2.9)	1

ACEI, angiotensin-converting enzyme inhibitor; ARB, angiotensin receptor blocker; ARNI, angiotensin receptor neprilysin inhibitor

There were no significant differences in antiplatelet drug applications other than aspirin (clopidogrel or ticagrelor, p = 0.536) between the two groups. The proportions of young and older patients treated with ACEI/ARB/ARNI were similar (p > 0.05). Compared with older patients, younger patients complied better to β-blockers (92.6% vs. 80.1%, p = 0.008), but poorly to statins (93.5% vs. 97.9%, p = 0.038).

### Predictors of cardiac events

**Fig. 1:**
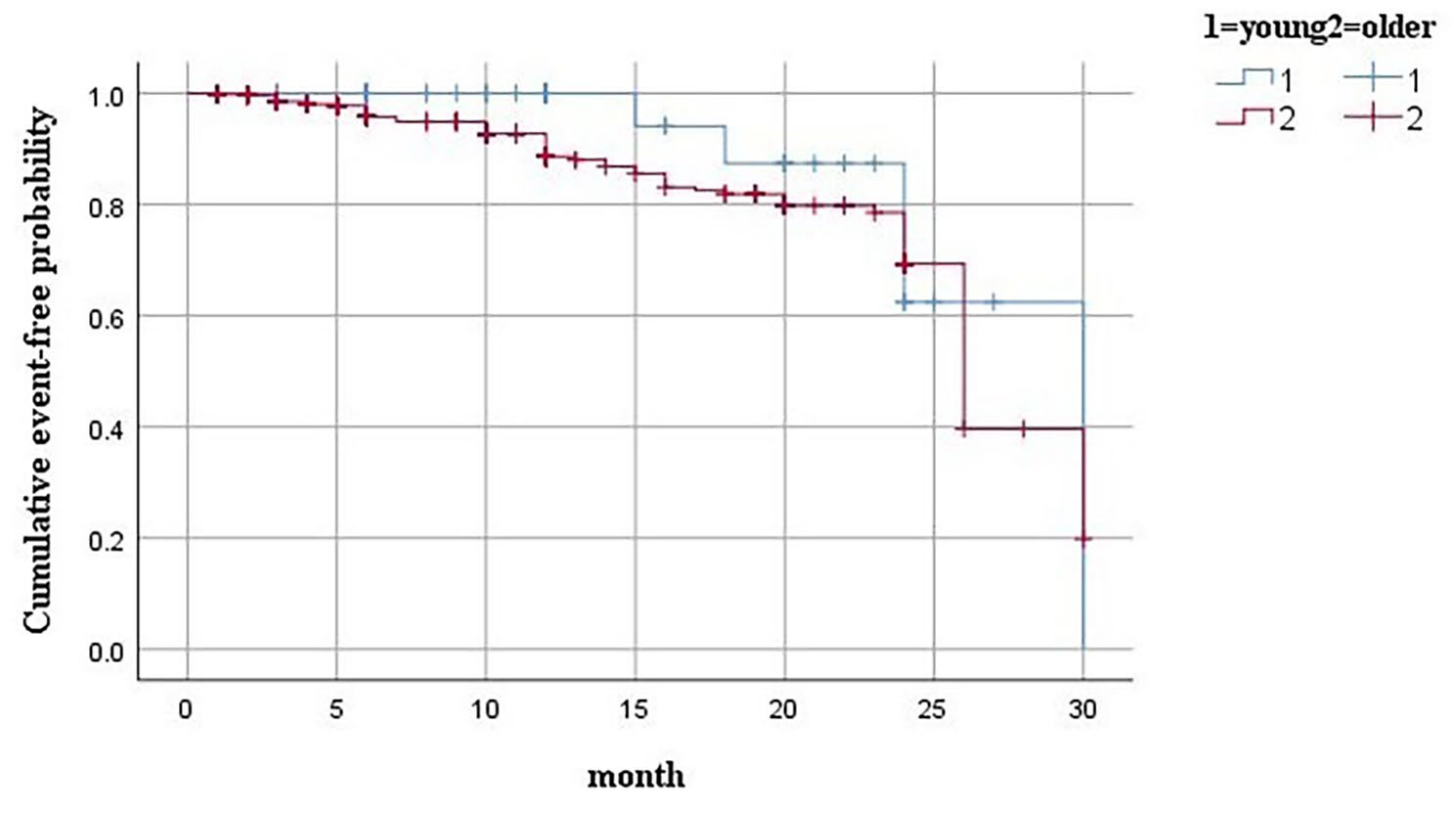
Kaplan-Meier curves for MACE of follow-up in two groups

The differences in clinical manifestations between the cardiac event and non-cardiac event groups are shown in Table 4. Figure 1 shows the Kaplan–Meier curves comparing all cardiovascular events in the younger and older patients with STEMI. Diabetes mellitus was the most significant predictor of MACE in the two groups (OR: 6.4, 95% CI: 1.219–33.593, p = 0.045; OR: 1.802, 95% CI: 1.087–2.985, p = 0.029), while sex (OR: 2.2, 95% CI: 1.323–3.656, p = 0.003) and age (68.45 ± 9.07 vs. 64.07 ± 9.72, p < 0.001) were also associated with MACE in the older group. (Fig. 1 Kaplan-Meier curves for MACE of follow-up in two groups)

**Table 4:** Relative risk for recurrent cardiac events during follow-up period in the two groups on logistic regression

	Age ≦ 45 years			Age > 45 years		
Variables	OR	95% CI	P value	OR	95% CI	P value
Sex(female)	7.5	0.518–108.45	0.223	2.2	1.323–3.656	0.003
Hypertension	3.406	0.748–15.506	0.127	1.438	0.858–2.411	0.201
Diabetes mellitus	6.4	1.219–33.593	0.045	1.802	1.087–2.985	0.029
Smoker	2.245	0.258–19.573	0.673	1.029	0.505–3.363	0.527
Drink alcohol	1.281	1.23–4.418	0.142	1.082	0.628–1.864	0.78
Family history of IHD	1.25	1.112–1.405	0.338	2.095	1.024–4.288	0.047

IHD, ischaemic heart disease; OR, odds ratio; CI, confidence interval

## Discussion

So far, there has been no standard definition of “young” age in patients with STEMI. In most studies, the cutoff of age were set at 40–50 years old (no more than 55), [11–13]. According to the criterion of the United Nations, [14] which defined over 45 years as older adulthood or above, so we choose 45 years as thresholds of age between young and older patients. In this study, 15.4% of patients with STEMI were aged less than 45 years, indicating an increasing incidence of AMI in young adults in China. The young patients with STEMI were more of males (98.1%), smokers (76.9%), and those with AUD (63.9%) or a family history of IHD (17.6%). However, it is not surprising that lower rates of hypertension and diabetes were observed in younger patients because the incidence of these metabolic risk factors increases with age. Studies have shown that smoking is the predominant cardiovascular risk factor in young patients, which could explain this increased trend in young Chinese patients over the last 10 years, an estimated 72% of Chinese individuals over the age of 15 have been exposed to second-hand smoke [15]. An observational study conducted in the United States showed that the prevalence of marijuana and cocaine use (which have the same effect as nicotine in cigarettes) in patients with AMI under 40 years of age is higher [16]. Besides leading to lung disease, nicotine could aggravate the degeneration of blood vessel smooth muscle cells by inducing an inflammatory response (via the mitogenactivated protein kinase pathway) and increasing oxidative stress for NADPH oxidase-1, thereby accelerating arteriosclerosis and plaques instability, which was related to ST-segment elevation myocardial infarction [17–21]. It has been shown that young patients are more likely to present with LAD artery-related myocardial infarction, and similar to the results of other studies [3], the independent risk factor was smoking in this study.

Western young patients have higher LDL cholesterol and triglyceride levels than Chinese patients [3]. Non-HDL cholesterol is more strongly associated with premature coronary heart disease in young Western patient populations than in the Chinese population [22]. However, similar to a previous study in the West, the proportion of abnormal blood lipids in young patients was higher than that of older patients in this study—that is, higher levels of TG, TC, and LDL and lower levels of HDL. Therefore, guidelines suggest that young people need to maintain a healthy diet and exercise regularly. Interestingly, uric acid levels have been shown to be associated with endothelial dysfunction, inflammation, and thrombosis, [23] indicating the instability of coronary plaques. However, the association between serum uric acid levels and cardiovascular diseases (CVD) may not be J-shaped. Previous studies have reported that an approximate U-shaped association between serum uric acid levels and 10-year CVD risk scores exists in men, while a J-shaped association existed in women [24]. Uric acid levels were obviously higher in young patients with STEMI in this study than in older patients, although the value did not exceed the upper limit. This result may be explained by the higher rate of AUD in patients under 45 years of age and the subsequent reduced water intake.

Similar to the result of a previous study, the results of this study showed that the most culpable vessel was the LAD artery in younger patients and the right coronary artery in older patients [25]. The reason for this was uncertain; however, smoking was an important factor in the younger group in this study. Almost one-third (35.1%) of young patients with STEMI in this study had multi-vessel disease; however, the proportion was obviously lower than that in the older group, which could be related to the worse mortality outcomes observed in a previous study [26].

The mortality of patients in the two groups was very low because the follow-up time was not protracted, and cardiovascular events were the main outcome in this study. Some studies have reported that younger patients with STEMI had higher mortality during follow-up. Diabetes was also shown in this study to have a strong trend towards MACE in both young and older patients with STEMI. Age and sex were important predictors of adverse events in older patients. Family history of IHD is a risk factor for young myocardial infarction, however, it may not be directly related to recurrent cardiovascular events, which can be attributed to anterior myocardial infarction, older and diabetes [26, 27]. Smoking cessation within 1 year after myocardial infarction was associated with more than a 50% lower all-cause and cardiovascular mortality in a previous study with a median follow-up of 11.2 years [28]. Persistent smoking may be a powerful predictor for the recurrence of cardiac events in young patients with STEMI [3, 29]. The same relationship was not indicated in this study because fewer people enrolled. However, in addition to smoking cessation, more attention should be paid to blood glucose monitoring in young patients with STEMI.

This study has several important limitations. First, as a retrospective analysis, our work was restricted to populations in the western region of Shandong Province. Second, eating habits may have affected the levels of blood lipids and uric acid. Third, in this study, only patients with acute coronary syndrome who required percutaneous coronary intervention were evaluated, so the number of patients was just 701 in two years, and although the data could be accurately obtained from medical records, it could also have led to statistical bias and deviation. In addition, the long-term prognosis of STEMI, especially mortality, was not obtained during the short follow-up period, and the relationship between traditional cardiovascular risk factors such as smoking and adverse events may be incorrect. Finally, although covariates were adjusted in the multivariate regression models in this observational study, other confounders might exist and may have led to incorrect conclusions.

In conclusion, For young people especially with a family history of IHD, realizing and managing the level of serum uric acid is also important, besides abstaining from smoking, drinking and dyslipidemia. Based on the results of this study, we suggest that diabetes should be treated to reduce the future events in young patients. Larger studies are warranted to include more patients and longer follow-up periods, and the results may be different. As the research population increases, age groups can be divided by a 10-year interval (such as 30–40/41–50/51–60), which may lead to more interesting results.

## Data Availability

The data will be shared on reasonable request to the corresponding author.
